# Filaggrin gene variants among Saudi patients with ichthyosis vulgaris

**DOI:** 10.1186/s12920-023-01700-x

**Published:** 2023-10-23

**Authors:** Omar Mohammed Alakloby, Fatimah Almuqarrab, Johannes Zschocke, Mathias Schmuth, Adnan Abdulkareem, Kholood Alnutaifi, Francis Borgio, Robert Gruber, Hans Christian Hennies

**Affiliations:** 1 Department of Dermatology, College of Medicine, Imam Abdulrahman Bin Faisal University, Dammam, Saudi Arabia; 2 Dermatology Department, King Saud University Medical City, Riyadh, Saudi Arabia; 3 Institute of Human Genetics, Medical University of Innsbruck, Innsbruck, Austria; 4 Department of Dermatology, Medical University of Innsbruck, Innsbruck, Austria; 5 King Saud Medical City, Riyadh, Saudi Arabia; 6 Department of Genetic Research, Institute for Research and Medical Consultations (IRMC), Imam Abdulrahman Bin Faisal University, Dammam, Saudi Arabia; 7 Department of Biological and Geographical Sciences, University of Huddersfield, Huddersfield, UK; 8 Cologne Center for Genomics, University Hospital Cologne, Cologne, Germany

**Keywords:** Ichthyosis vulgaris, Gene mutation, Filaggrin, Atopic diathesis, Atopic dermatitis, Ichthyoses

## Abstract

Ichthyoses are a heterogeneous group of cornification disorders. The most common form of ichthyoses is ichthyosis vulgaris (IV) ([OMIM] #146,700), which can be inherited as autosomal semi-dominant mutation in the filaggrin gene (*FLG*). We present the findings of a study involving 35 Saudi patients with a clinical diagnosis of ichthyosis vulgaris. For identifying the pathogenic mutation of their disease, we used Sanger sequencing analysis of the extracted DNA samples. We also identified the underlying 22 *FLG* variants, which have been seen before. However, the detected mutations do not involve the common p.R501* c. 2282del4 mutations reported in European populations. Indeed, we did not identify any statistical influence of the homozygous or heterozygous genotypes on the phenotype severity of the disease.

## Background

Ichthyoses are a heterogeneous group of cornification disorders characterized by thickening of the stratum corneum, scaling, xerosis, and compromised perspiration. It can be classified into either a nonsyndromic or syndromic disease based on whether it is confined to the skin [1]. Several authors have developed classification systems based on genetic mutations, the mode of inheritance, clinical findings, the concept of retention versus hyperproliferative keratosis, or based on biochemical information [2–5].

The most common form of ichthyoses is ichthyosis vulgaris (IV) ([OMIM] #146,700), which is inherited in an autosomal semi-dominant manner [6, 7]. Indeed, the filaggrin (filament aggregating protein, *FLG*; #135,940) gene can be mutated in patients with IV. The gene *FLG* encodes a 400 kDa histidine-rich protein consisting of 10–12 repeats, each comprising 324 amino acids and short linkers, that is essentially involved in the cornification process of the cornified cell envelope [8–11]. Notably, the mutated gene is mapped to chromosome 1q21 within a cluster of markers in the epidermal differentiation complex region [12, 13]. The gene comprises three exons: exon one is noncoding, exon 2 contains the translation initiation codon, and exon 3 encodes a significant part of the profilaggrin protein [14].

In 2002, Compton et al. [12] mapped IV in a multi-generational family to the epidermal differentiation complex, including *FLG*. In 2006, Smith et al. identified a homozygous p. R501* mutation and p. R501*/c. 2282del4 compound heterozygous mutations in *FLG* in 15 families with a severe IV phenotype. The *c. 2282del4* mutation they identified leads to a premature termination codon, stopping *FLG* translation. Screening IV families demonstrated high frequencies of these variants in patients of European ancestry [8].

Furthermore, in 2006, Palmer et al. showed that the two loss-of-function *FLG* variants, p. R501* and c. *2282del4*, strongly predispose patients to atopic dermatitis. Atopic diathesis association with ichthyosis vulgaris is well established, as approximately 50% of IV patients develop atopic dermatitis [15–17].

Moreover, in 2006, Sandilands et al. described 15 variants of *FLG* to facilitate the genetic analysis, where European variants are either non-prevalent or rare. These variants they identified have resulted in a loss of *FLG* function [18].

In another study in 2007, Nomura et al. reported two other *FLG* variants, p. S2554* and c. *3321del*A [19].

This study describes the clinical and genotypic data of 35 Saudi patients diagnosed clinically with ichthyosis vulgaris. Notably, we identified 22 common *FLG* variants mutated in our study population, all of which were missense variants that have been seen before.

## Materials and methods

### Ethical compliance

This descriptive-analytic study involving human participants obtained ethical approval from the Institutional Research Board of Imam Abdulrahman Bin Faisal University, Dammam. All study participants signed written informed consent forms.

### Patients and phenotypes

Saliva samples were collected from 35 individuals diagnosed clinically with ichthyosis vulgaris by experienced dermatologists. The Oragene^TM^.DNA collection kit (OG-500 Disc format, DNA Genotek Inc, Ottawa, Ontario, Canada) was used for collecting saliva samples by trained nurses.The patients were asked to fast for 30 min before the collection. Fifteen minutes before sample collection, the patients were asked to rinse their mouths with water. The patients were then asked to rub the inside of their mouths with their tongues for 15 s and to spit the saliva into an empty container until the amount of liquid saliva (not the bubbles) reached the level shown on the collection container. After that, the container was sealed, labeled, and gently shaken for 10 s to mix the saliva with the Oragene solution. For the DNA extraction, a standard quantity of 2.0 ml Oragene/saliva mixture was used as the manufacturer’s protocol recommended.

A standard form was prepared for collecting patient data, including age, sex, ethnicity, parents’ consanguinity, coexisting atopy, degree of scaling and skin dryness, and inheritance pattern according to the family pedigree.

### Genotyping

Genomic DNA was extracted using prepIP-L2P (DNA Genotek, Ottawa, ON, Canada) per the manufacturer’s protocol, and purity and quantity were checked using NanoDrop 8000 (Thermo Fisher Scientific Inc., Massachusetts, United States). PCR overlapping method was used to amplify target exons using conventional cycle settings and in-house developed primers. Regions of *FLG* gene were amplified individually in Bio-Rad MyCycler™ (Bio-Rad, Berkeley, CA, USA) (95 °C/4 minutes; 30 cycles of 95 °C/1 minute; annealing/1 minute; 72 °C/1.5 min; and final extension 72 °C/5 minutes) using amplification primers listed in Table 1, with AmpliTaq Gold DNA Polymerases (Thermo Fisher Scientific Inc., Massachusetts, United States). All the primers were synthesized from Applied Biosystems (Life Technologies Corporation, USA). The purified (QIAquick PCR Purification Kit, Qiagen, Germany) amplicons were cycle sequenced with forward and revere primers separately, as listed in Table 1, using a BigDye Terminator Cycle Sequencing Kit (Life Technologies Corporation, USA). The purified cycle sequenced products from forward and revere primers separately were sequenced using POP 7 in a Genetic Analyzer 3500 (Life Technologies Corporation, USA).

**Table 1:** List of primers designed and used to amplify FLG gene

	Name of the primer	Sequence	Amplified PCR product	Annealing temperature
**1**	FLG501/2282F	AGCCACCAAGAGTCCACACGTGGC	971	72 °C
**2**	FLG501/2282R	TGATGGTGACCAGCCTGTCCATGGCC		
**3**	FLG2447F	CGGTCAGCAGGAAGGTCTG	487	65 °C
**4**	FLG2447R	ATCCCCAGTTCCTGCTTGTC		
**5**	FLG501F	ACCAAGAGTCCACACGTGGCCG	224	70 °C
**6**	FLG501R	TCTGCTTGACCCCGGGTGTCCAC		
**7**	FLG2282F	GACAAGCTTCATCTGCAGTCAGAGA	203	63.6 °C
**8**	FLG2282R	GTAGAGGAAAGACCCTGAACGTCG		

The DNA sequences obtained through Sanger Sequencing for all the patients were analyzed with SeqmanPro (DNASTAR Inc., Madison, WI, USA) [20]. Unknown missense variants were considered possibly pathogenic if they were anticipated to be harmful by at least two of the algorithms MutTaster [21], SIFT [22], and PolyPhen2 [23], impacted highly conserved amino acids and were not discovered as homozygous variants in control DNA sequences(www.internationalgenome.org). Human Splicing Finder 3.0 examined splice site variants [24]. PFAM was used to identify protein domains [25].

### Statistical analysis

The collected data were entered into a computer database and analyzed statistically using Excel software [26]. We used the chi-square test for performing a logistic regression analysis to examine the influence of our descriptive genotype variables (homozygosity or heterozygosity of the *FLG* variant) on each of the phenotype variables severity (scale severity, palmer hyper linearity, and pruritus). The chi-square was calculated using the formula:

*χ*²= ∑ [(observed frequency – expected frequency)²/expected frequency].

## Results

### Patient characteristics

**Fig. 1:**
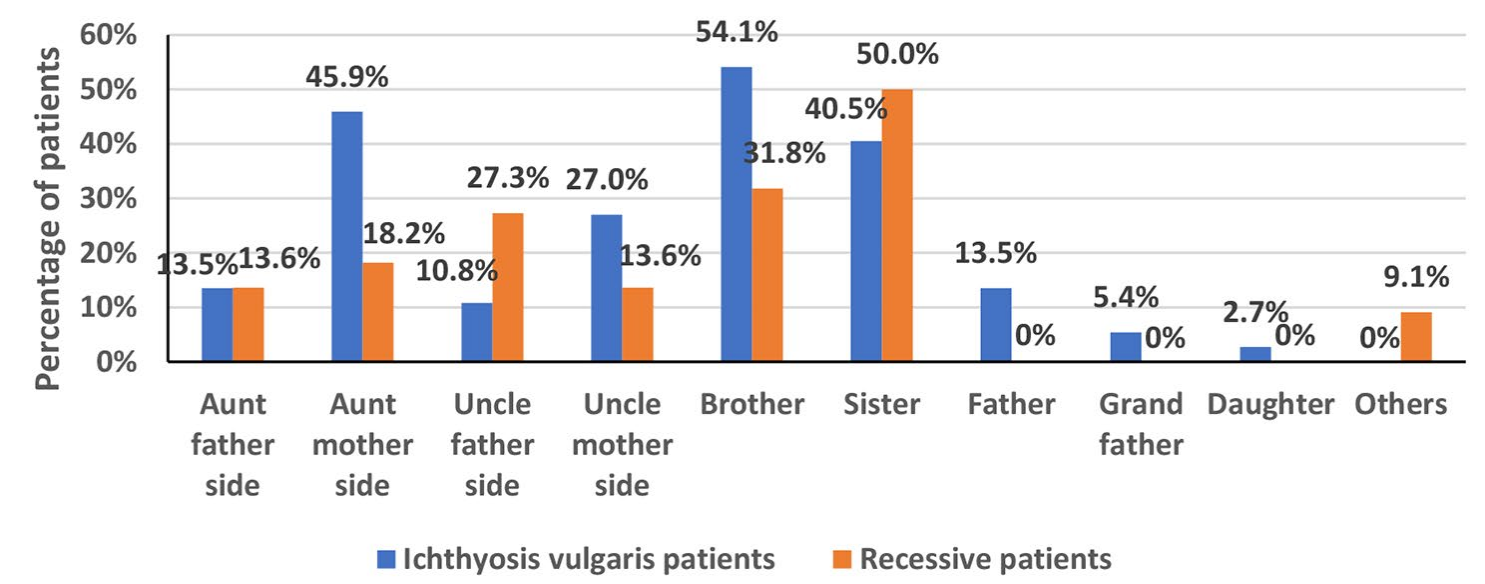
Other affected family members

**Fig. 2:**
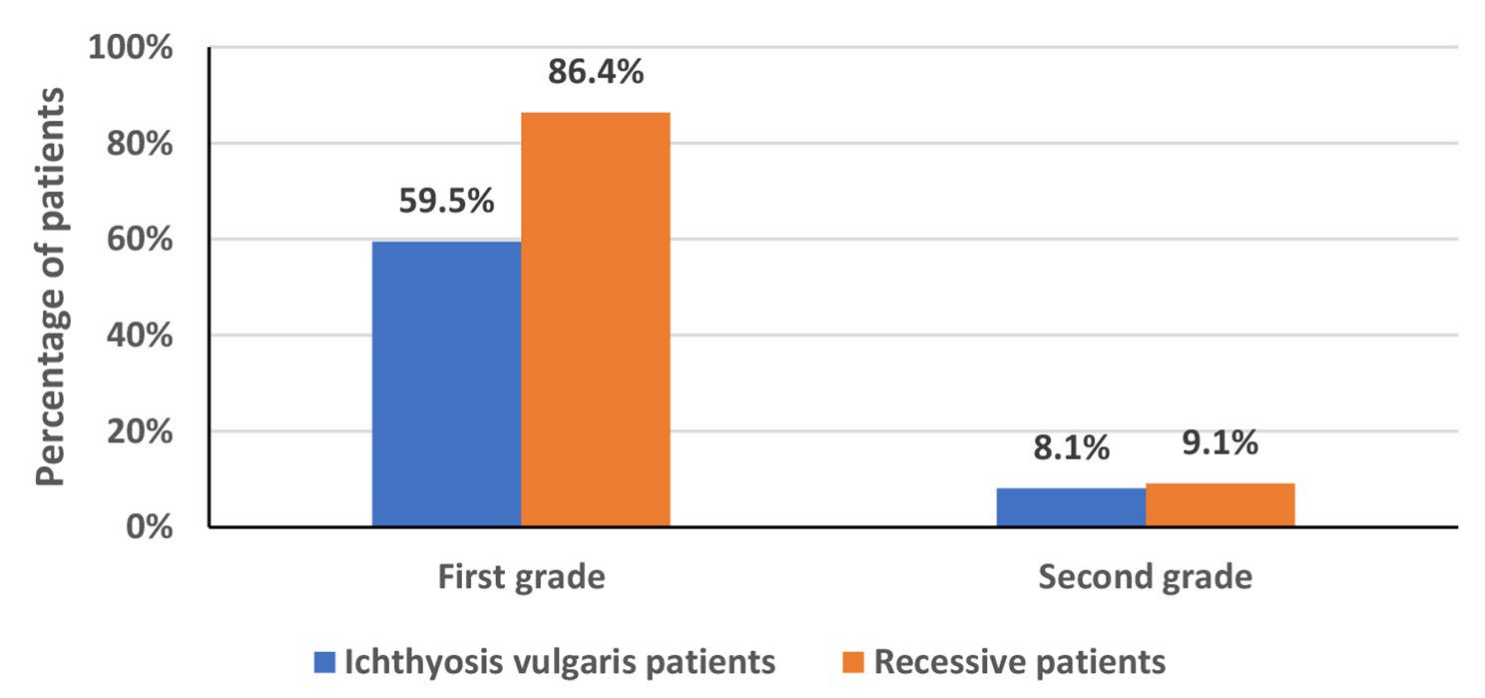
Parent consanguinity

A total of 35 patients with ichthyosis vulgaris were included in this study; 22 (62.9%) were males, and 13 (37.1%) were females, giving a male-to-female ratio of 1.7:1. All patients were Saudis; their mean age was 21.1 ± 13.3. All patients’ symptoms presented after ten weeks of birth. There were 25 (68%) consanguineous marriages among the parents. Thirty-two (91.9%) patients had other affected family members with IV. (Figures 1 and 2)

### Clinical findings

All patients had Fitzpatrick skin color type 4 and mild to moderate skin dryness. The majority of our patients had fine scales. The scales vary in severity and colors from delicate light brown in 84.5% of patients to coarse, large brown in 15.15%; most patients (84.8%) had scales on the neck, trunk, or extremities, while only a minority of them (5.4%) showed a generalized distribution of the scales. Flexures were spared in 20 (57%) patients. 40% of our patients had dental abnormalities, either in the form of caries (28.5%), abnormal occlusion (2.8%), or a combination of both (8.5%).

Plantar keratoderma was seen in 18 (51.2%) patients and palmoplantar keratoderma was seen in 4 (11.4%). Palmar hyperlinearity was encountered among 28 (80%) IV patients, while pruritis was discovered in 14 (40%). Their demographic data are outlined in Table 2.

**Table 2:** Demographical and clinical characteristics of Ichthyosis vulgaris patients (Total = 35)

Demographical characteristics		n (%)
**Sex**		
Male		22 (62.86)
Female		13 (37.14)
**Age**		
**Mean age**	21.5 ± 13.3	
< 10		4 (17.14)
10–20		13 (37.14)
> 20		18 (51.4)
**Fitzpatrick skin type**		35 (100)
**Affected Family Members**		32 (91.4%)
**Consanguinity of parents**		
First degree		22 (62.8)
Second degree		3 (8.5)
None		10 (28.5)
**Fitzpatrick skin type**		
Type 4		35 (100)
**Coexisting abnormality**		
Sickle cell anemia		2 (5.7)
Others		2 (5.7)
Pruritus		14 (40)
Allergic rhinitis		1 (2.9)
Other allergies		1 (2.9)
**Oral retinoids**		
Acitretin		3 (8.6)
**Oral retinoid dose per day**		
5–10 mg		2 (66.7)
21–30 mg		1 (33.3)
**Oral retinoids duration**		
1–3 months		1 (33.3)
3–4 years		2 (66.7)
**Topical therapy**		
Moisturizing		33 (94.3)
Keratolytic and moisturizing		1 (2.9)
**Skin dryness**		
Mild		20 (57.1)
Moderate		15 (42.9)
**Scaling**		
Mild		25 (71.4)
Moderate		10 (28.6)
**Scaling scales**		
Fine		29 (82.9)
Coarse and large		6 (17.1)
**Scaling color of scales**		
Light brown		26 (74.3)
Brown		3 (8.6)
White		6 (17.1)
**Scaling site of scaling**		
Neck, trunk, or extremities		35 (100)
Sparing flexures		20 (57.1)
Keratosis pilaris		1 (2.9)
**Hyperkeratosis**		
Plantar		18 (51.4)
Palmoplantar		4 (11.4)
Palmar hyperlinearity		28 (80)
**Teeth**		
Caries		9 (25.7)
Abnormal occlusion and caries		2 (5.7)
Abnormal occlusion		1 (2.9)
Abnormal occlusion, caries, and discoloration		1 (2.9)
Caries and discoloration		1 (2.9)
**Saliva collection**		35 (100)
***FLG*** **Variant**		22

Non-cutaneous coexisting disorders were identified in a minority of the patients, including sickle cell anemia (5.7%) or G6PD deficiency (2.8%). However, they might represent a confounding factor.

Most of our patients were treated with topical measures and were well-controlled; however, 8.5% required systemic treatment. We treated those patients with oral acitretin (neotigason capsules, Teva UK), whereas 66.6% of the treated patients required low-dose acitretin ranging between 5 and 10 mg/day, and one patient needed a higher dose of acitretin 20–30 mg/day for their disease control. The decision to use low-dose systemic retinoids versus higher doses is based on the symptomatology and severity of the clinical findings. Patients with milder, fine asymptomatic scales and palmoplantar keratosis were treated with low-dose retinoids, while those with pruritic larger, coarser scales were treated with higher doses. Fortunately, two patients responded well to systemic retinoids and tolerated continued use for over three years. The scale severity, hyperkeratosis thickness, and pruritus were improved with the treatment use. Topical treatments were given alone for minor cases or as an adjuvant to systemic therapies for severe patients. The topical choices were selected based on the involved areas, the type of scales, the degree of dryness, and the presence or absence of symptomatology. Patients with scalp involvement were treated with coal tar (5%)/salicylic acid (2%) shampoo, patients with dry, scaly skin were given Akerat cream (urea/salicylic acid), and patients with pruritus were advised to use LIPIKAR BAUME AP + from LA ROCHE for sensitive skin. The patients responded to the provided regimens, resulting in an improved quality of life and reasonable control of their clinical presentation.

### FLG genetic diversity of the patients with IV

We comprehensively sequenced the gene *FLG* using the overlapping PCR method. No genetic alterations were identified in exons 1 and 2. We identified 22 different variants in exon 3; all have been reported previously with a known allele frequency according to the Genome Aggregation Database (gnomAD) [27]. (Table 3).

All variants found here were either missense or silent variants. We did not find the known loss-of-function variants p.R501*, and *c. 2282del4* in our samples. The most prevalent allele size in our Saudi patient sample was 7–8 repeats. The two most frequent *FLG* variants were *p.H2507Q* and *p.G2545R*, presenting in 48.5% of our patients. Three identified variants were silent variants (#s 2, 3, 20) that might have no impact on the disease (Table 3). However, we do not have data about their allele frequencies in the general Saudi population.

## Discussion

The gene *FLG* consists of 3 exons. Exon 3 is the largest and is the coding segment of all filaggrin repeats. In 2006, Smith et al. [8] detected the nonsense mutation p. R501* near repeat 1. They further sequenced the *FLG* leading to the identification of a second mutation, c. *2282del4*, causing premature termination codon [8]. In 2007, Nomura et al. [19], on the other hand, screened Japanese families, however, only some Japanese individuals with IV carried the European mutations. Complete sequencing of *FLG* identified two other mutations in the Japanese IV families, namely, p.S2554* and c.*3321delA*. Similarly, in 2014, Polcari et al. [28] reported a low prevalence of European-specific mutations in the African American population. In parallel with the Japanese and the African American populations, ours does not have the common European mutations.

Near 60 *FLG* null mutations have been identified in European and Asian populations; some are unique to an ancestral population, and different ancestries share others [29, 30].

In 2018, Hassani et al. [31]. sequenced the entire *FLG* in Iranian patients and identified 45 variants with two previously unknown variants. None of the identified *FLG* variants were loss-of-function variants and were supposed to be non-pathogenic (nonsense). Considering the frequency of repeat numbers of *FLG* and the identified DNA variants between the patients and the control group, they concluded that other mechanisms, possibly inflammatory-driven or epigenetic *FLG* functional deficiency might be involved in the pathogenesis of IV rather than the *FLG* nonsense mutation [31].

In line with the Iranian study, sequencing the entire *FLG* coding repeats identified 22 *FLG* variants. In fact, most of the identified *FLG* variants were missense with a known general allele frequency; however, six were silent variants. Furthermore, we tested the hypothesis of correlating the *FLG* variants to the clinical severity of our IV patients. To correlate the genetic to the phenotype severity, we analyzed three clinical factors: scaling severity, palmer hyperlinearity, and the presence of pruritus. In contrast to studies of loss-of-function *FLG* variants, we did not identify any influence of the *FLG* variants on the clinical severity of the disease (Table 4).

**Table 3:** DNA variants in *FLG* in Saudi patients with ichthyosis vulgaris

#s	DNA change [NM_002016.2(FLG)]	Amino acid change [NP_002007.1]	SNP (NCBI)	Genotype frequency	Alternate allele frequency (gnomAD)	
1	*c.7330 A > G*	*p.L2444Glu*	rs71625200	14 (40%)	0.262	
2	*c.7371G > A*	*p. Glu2457Asp*	rs112771575	4 (11.4%)	0.000435	
3	*c.7398G > A*	*p.Pro2466=*	rs71625199	4 (11.4%)	0.00000795	
4	*c.7442T > C*	*p.Leu2481Ser*	rs55650366	15 (42.8%)	0.262	
5	*c.7452 A > G*	*p.Arg2484Ser*	rs111937188	4 (11.4%)	0.000212	
6	*c.7521 C > G*	*p.His2507Gln*	rs3126074	17 (48.5%)	0.297	
7	*c.7552 C > G*	*p.Arg2518Gly*	rs111618175	4 (11.4%)	0.000442	
8	*c.7557 C > T*	*p.Asn2519Lys*	rs113878714	4 (11.4%)	0.000046	
9	*c.7633G > A*	*p.Gly2545Arg*	rs3126072	17 (48.5%)	0.300	
10	*c.7387 C > G*	*p.His2463Asp*	rs113188294		0.00801	
11	*c.2263G > A*	*p.Glu755Lys*	rs74129461	5 (14.2%)	0.256	
12	*c.2225 C > A*	*p.Ser742Tyr*	rs3120654	5 (14.2%)	0.036	
13	*c.2224T > C*	*p.Ser742Pro*	rs774808102	1 (2.8%)	0.0000119	
14	*c.2174 C > T*	*p.Thr725Ile*	rs3120655	5 (14.2%)	0.0343	
15	*c.2035 A > C*	*p.Lys679Gln*	rs113685999	1 (2.8%)	0.0011	
16	*c.1992 C > A*	*p.His664Gln*	rs113652604	2 (5.7%)	0.0101	
17	*c.1949 A > G*	*p.Gln650Arg*	rs111791016	1 (2.8%)	0.000456	
18	*c.1819 C > G*	*p.Gln607Lys*	rs113473008	1 (2.8%)	0.00000795	
19	*c.1791 C > G*	*p.Ser597Ar*	rs112462459	1 (2.8%)	0.000442	
20	*c.1737 C > T*	*p.Asp579=*	rs145104949		0.000647	
21	*c.1450 C > T*	*p.Arg484Trp*	rs113136594	1 (2.8%)	0.00000398	
22	*c.1432 C > T*	*p.Pro478Ser*	rs11584340	7 (20%)	0.262	

**Table 4:** Logistic regression analysis to determine the influence of the *FLG* genotype on the phenotype severity

	Scaling severity	Palmoplantar hyper linearity	Pruritus
Chi square	2.5	1.4	3.5
Critical value	5.99	5.99	5.99
**p-value**	0.54	0.69	0.3

## Conclusion

In conclusion, we identified missense and silent *FLG* variants, but no loss-of-function variants in our Saudi patients with IV. This is in contrast to studies from several different ethnic groups. Moreover, there was no influence of the detected *FLG* genotype on the phenotype severity of the disease. This study may serve as a Saudi reference variant spectrum for the *FLG* gene mutation in ichthyosis vulgaris patients. In the future, we hope to perform more national cohort studies and further analyze the newly reported variants by comparing them with a control group.

## Data Availability

The datasets used and analyzed during the current study are available from the corresponding author upon reasonable request.
